# Presymptomatic diagnosis of Fabry’s disease: a case report

**DOI:** 10.1186/s13256-016-1124-z

**Published:** 2016-11-29

**Authors:** Rasmus Bo Hasselbalch, Per Lav Madsen, Henning Bundgaard, Juliane Theilade

**Affiliations:** 1 Department of Cardiology, Copenhagen University Hospital – Herlev-Gentofte Hospital, Herlev Ringvej 75, Herlev, 2730 Denmark; 2 Department of Cardiology, Copenhagen University Hospital – Rigshospitalet, Blegdamsvej 9, 2100 Copenhagen, Denmark

**Keywords:** Fabry’s disease, Late onset, Inherited cardiac diseases, Family testing

## Abstract

**Background:**

Fabry’s disease is a rare X-linked genetic disorder characterized by reduced levels of the α-galactosidase A enzyme. It may present with a cardiac phenotype resembling hypertrophic cardiomyopathy. However, as a specific enzyme replacement therapy is available, it remains an important differential diagnoses in patients presenting with cardiac hypertrophy. In boys, onset has been reported in early childhood with complaints initially comprising neuropathic pain, reduced sweat production, and gastrointestinal symptoms. Later the cardiac, renal, and central nervous systems may become affected. Female mutation carriers may remain asymptomatic or present at a later age with varying symptoms and clinical manifestations due to random inactivation of the X chromosome in different organs.

**Case presentation:**

Here we present a case of Fabry’s disease diagnosed in the daughter of an elderly, Caucasian woman (81 years old) with late-onset cardiac conduction disease and heart failure. We discuss the implications of cascade screening relatives of elderly probands.

**Conclusions:**

Irrespective of the patient’s age, physicians must be on the lookout for phenocopies when identifying patients with possibly inheritable cardiomyopathies. The specific – precise – diagnosis may be crucial for the patient as well as the relatives.

## Background

Fabry’s disease (FD) is a rare X-linked lysosomal storage disease with an estimated prevalence of 1:117,000 [1]. Numerous private mutations in the gene encoding for the α-galactosidase A enzyme (GLA) have been associated with the condition. The mutations result in defect catabolism of glycosphingolipid resulting from lowered activity of the lysosomal α-galactosidase A enzyme. Affected individuals accumulate glycosphingolipids in the lysosomes and cytoplasm of cells throughout the body [2]. Typical early symptoms include temperature-provoked pain in the extremities, reduced ability to sweat (hypo- or anhydrosis), nausea, and abdominal pain. Later arterial hypertension, left ventricular (LV) hypertrophy, heart failure, conduction abnormalities, renal insufficiency, and symptoms from the central nervous system may present (stroke, tinnitus, and hearing impairment). The presenting symptoms usually manifest during childhood but can be delayed especially in heterozygous cases. Patients with FD may gradually develop terminal renal insufficiency and severe cardiac dysfunction and their life expectancy is reduced by 20 and 15 years in males and females, respectively [2]. A cardiac variant, characterized by only – or mainly – cardiac involvement has been reported [3]. This case report reminds us that Fabry’s disease can cause familial cardiomyopathy that may present late and/or in a milder form in female mutation carriers.

## Case presentation

### The mother (patient 1)

A 74-year-old Caucasian woman was referred due to gradual worsening of dyspnea with aggravation during a recent skiing trip. Her previous medical history included mild asthma and osteoporosis, surgical treatment for rectal cancer 30 years prior to presentation, and an evaluation for dizziness 4 years previously when a computed tomography scan of the cerebrum was without any positive findings.

The patient denied palpitations but reported occasional retrosternal oppression and two incidences of near syncope. Aside from age, she had no risk factors for ischemic heart disease.

A physical examination revealed an arterial blood pressure of 132/70 mmHg, a heart rate of 40 bpm, normal oxygen saturation, normal body temperature, and no dyspnea at rest. Further examination revealed jugular vein distension while sitting upright in bed and a discreet systolic murmur. The electrocardiogram (ECG) showed atrial fibrillation and complete atrioventricular block. Blood tests were normal except from consistent low-grade elevation of the cardiac troponin I. An echocardiogram showed severely dilated left atria, mild mitral regurgitation, borderline dilated LV with near-normal systolic function and no hypertrophy. Acute coronary syndrome was not considered present and our patient underwent an uneventful implantation of a dual-chamber pacemaker (DDDR). Following discharge, the patient was followed by her private cardiologist. It was later noted that cardiac hypertrophy and a slight decrease in LV ejection fraction had developed, the latter possibly considered secondary to cardiac pacing.

**Fig. 1:**
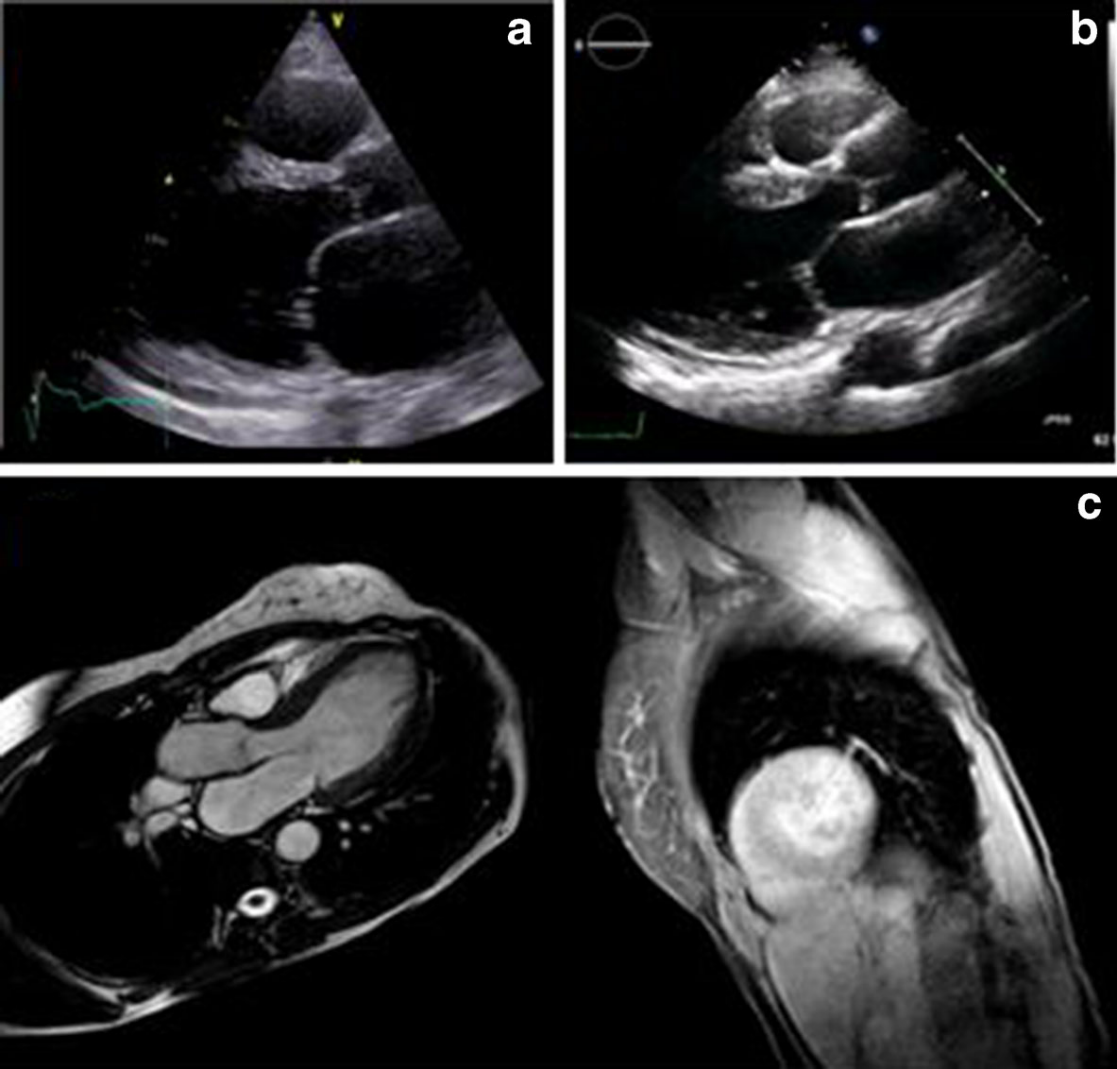
Echocardiograms from the two patients presented. Panel **a**, parasternal images from the asymptomatic daughter. The interventricular septum measured 15 mm (reference < 11 mm). Panel **b**, the echocardiogram from the mother aged 81 years. The scan demonstrates a dilated left ventricle (LVIDd 46 mm/m^2^, reference < 32 mm/m^2^) and severe systolic heart failure (left ventricular ejection fraction estimated to 15%). Panel **c**, cardiac magnetic resonance imaging of the daughter. The cardiac magnetic resonance scan demonstrated preserved systolic function and mild left ventricular hypertrophy (143 g/m^2^, normal range 69–141 g/m^2^). The cardiac magnetic resonance also demonstrated late gadolinium hyperenhancement in inferolateral left ventricular myocardial segments, that is, in a pattern highly suspicious of a cardiac phenotype with Fabry’s disease

Over the next 5 years, our patient was repeatedly admitted to the emergency department with dyspnea. At our institution, the echocardiogram was repeated when our patient, at the age of 78 years, was admitted with severe respiratory distress interpreted as pulmonary edema. Her LV ejection fraction had decreased to 15% (Fig. 1a). A subsequent coronary angiogram was without any coronary artery lesions and she was diagnosed with non-ischemic, non-valvular dilated cardiomyopathy (DCM). Subsequently, our patient’s pacemaker was upgraded to a biventricular pacemaker without anti-tachycardia pacing or defibrillator function. At age 81 years, our patient suffered several episodes of ventricular tachyarrhythmias and died within days. During hospitalizations from the pacemaker insertion, renal function had declined to an estimated glomerular filtration rate of 30 ml/min/m^2^ (normal range > 60 ml/min/m^2^).

### The daughter (patient 2)

**Fig. 2:**
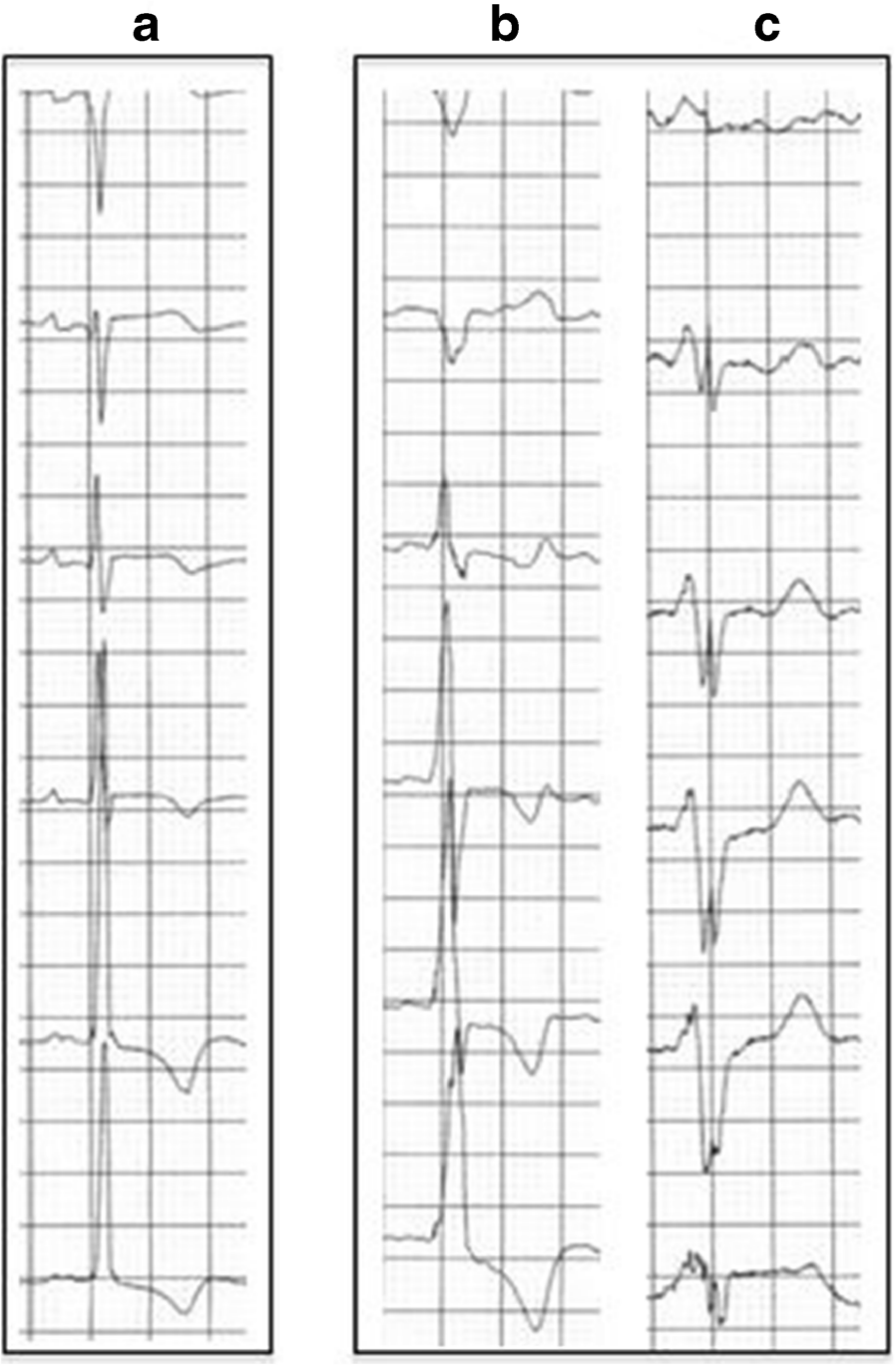
Electrocardiograms from the two patients presented. Panel **a**, electrocardiogram from the 52-year-old asymptomatic daughter. Panel **b** and **c**, electrocardiograms from the proband at age 79 and 81 years, respectively

The daughter of patient 1 was referred for evaluation from her general practitioner (GP). She had contacted her GP because of her mother’s death asking if her mother’s cardiac condition might have been an inherited disorder, which might indicate a risk for herself. She was 52 years old, worked full-time and was physically active without limitations. Her medical history was unremarkable except for well-controlled hypertension. Blood samples showed elevated cardiac troponins (troponin I was 121 ng/L, normal range < 40 ng/L) and an ECG suggested LV hypertrophy. These test results prompted referral to our clinic. Her blood pressure was 142/98 mmHg, heart rate was 57 bpm. An echocardiogram showed hypertrophy (15 mm) of the interventricular septum without LV outflow tract obstruction. No other pathologies were identified on the echocardiogram (Fig. 1b). The LV ejection fraction was normal. Further blood work demonstrated a phosphocreatinine kinase myocardial band (CK-MB) of 5.9 μg/L (normal range < 7 μg/L), slightly elevated lactate dehydrogenase (212; U/L; normal range 105–205 U/L) and B-type natriuretic peptide (616 ng/L; normal range < 450 ng/L). Creatinine, electrolytes, thyroid hormones, hemoglobin, cholesterol, and hemoglobin A1c (HgbA_1_c) were all normal. A 24-hour blood pressure monitoring showed a mean of 116/80 mmHg, and a cardiac stress test was performed without eliciting symptoms or signs of ischemia and a normal blood pressure response was recorded during exercise. A 24-hour ambulatory blood pressure test confirmed that the patient’s hypertension was well-controlled (average 116/80 mmHg) and a 24-h ambulatory ECG monitoring did not identify arrhythmias. We compared this patient’s ECG with her mother’s (Fig. 2) and noted that the alterations were quite similar, and for this reason an inheritable cardiac condition was suspected.

Following information from our patient, genetic testing, and a cardiac magnetic resonance (CMR) imaging scan was ordered (Fig. 1c). The genetic testing identified a previously reported FD causing mutation in the coding part of the GLA gene (c.901C>T,p.R301*). CMR confirmed LV hypertrophy and demonstrated late gadolinium hyperenhancement in inferolateral segments in a mid-myocardial location. The scan was in agreement with a diagnosis of FD, and our patient was referred to a tertiary center for evaluation for treatment with enzyme replacement therapy (ERT).

## Discussion

In the family described here, the sudden cardiac death (SCD) of the proband at age 81 years, prompted her daughter to request cardiac testing, leading to the diagnosis of Fabry’s disease. Evaluation of relatives (cascade screening) for familial cardiac diseases is straightforward when a well-defined phenotype is present in the proband. However, screening of relatives to SCD victims has a low yield that decreases with the age of the index case [4–6]. Therefore, the national Danish guidelines for SCD do not recommend cascade screening in relatives of older probands (>50 (−60) years) [7, 8].

In brief, this report describes a mother and daughter with structurally similar cardiac phenotypes. The mother presented with complete heart block followed by development of LV hypertrophy and finally by LV systolic failure. Had the patients’ completely asymptomatic daughter inquired whether we would recommend cascade screening and initiate clinical testing of her, current guidelines would have discouraged us from doing so [7]. However, investigations had already been performed and were abnormal. Thus, the ECG and echocardiogram demonstrated LV hypertrophy that indicated further testing on the suspicion of hypertrophic cardiomyopathy (HCM).

HCM is not uncommon (estimated prevalence is 1:500) [9], and its diagnosis should prompt the search for the specific etiology as specific therapy is available for certain phenocopies, that is, Fabry’s disease and carnitine transporter defect. In general, management in HCM consists of genetic counseling, symptomatic relief in cases with LV outflow obstruction, stroke prevention in patients with concurrent atrial fibrillation, and monitoring of risk factors for SCD with recommendations of prophylactic implantable cardioverter-defibrillator (ICD) therapy in high-risk patients [10]. However, a number of phenocopies, acquired as well as genetic, may mimic HCM but – in contrast to genetic HCM – may be amenable to specific therapies. Most commonly, such phenocopies would be significant LV hypertrophy caused by poorly controlled hypertension, aortic stenosis or athlete’s heart. More rarely, obesity, hemochromatosis, and storage or metabolic diseases may elicit cardiac hypertrophy. In the family presented here, FD was one of several differential diagnoses as the proband had presented with conduction abnormalities, myocardial hypertrophy with progression to a dilated cardiomyopathy phenotype with concurrent development of renal insufficiency.

Whereas males with FD generally present in childhood, women often present later and with milder and more variable symptoms [2]. Except for admittance due to stomach pains and late-presenting kidney failure after the cardiac arrest in patient 1, none of the non-cardiac manifestations of FD were reported by either patient. Another diagnostic clue may be a short PR interval in the ECG of patients with FD. However, this feature was not seen in either case presented here. Both mother and daughter showed continuously elevated cardiac troponins, which has previously been reported in FD cohorts and seems to be correlated to cardiac hyperenhancement as assessed by late gadolinium CMR [11]. This specific pattern may be useful in differentiating FD from HCM and other storage diseases [12]. CMR with late gadolinium demonstrates specific findings differentiating FD from HCM [13]. When managing families with possibly inheritable cardiac conditions it is recommended that patients consider whether adjustments should be made with regard to specific job functions, insurances and retirement plans and that this is done before any clinical tests are performed [10].

## Conclusions

To provide optimal patient care, irrespective of patient age - physicians must be on the outlook for phenocopies when identifying patients with possibly inheritable cardiomyopathies. X chromosome-linked diseases are often considered not to affect female carriers, but due to random inactivation they may nonetheless develop discrete and/or late presenting symptoms that require recognition and treatment.

## Abbreviations

CK-MB: phosphocreatinine kinase myocardial band
CMR: cardiac magnetic resonance
DCM: dilated cardiomyopathy
DDDR: dual chamber pacemaker
ECG: electrocardiogram
ERT: enzyme replacement therapy
FD: Fabry’s disease
GLA: α-galactosidase A
GP: general practitioner
HCM: hypertrophic cardiomyopathy
HgbA_1_c: hemoglobin A1c
ICD: implantable cardioverter-defibrillator
LV: left ventricle
LVIDd: left ventricular internal diameter in diastole
SCD: sudden cardiac death
